# Syndrome de la moelle attachée chez l'enfant: à propos d’un cas

**DOI:** 10.11604/pamj.2019.34.151.18344

**Published:** 2019-11-15

**Authors:** Luphin Hode, Sourou Bruno Noukpozounkou, Josué Dejinnin Georges Avakoudjo, Thierry Alihonou, Beaudelaire Romulus Assan, Séraphin Antoine Gbenou, Michel Armand Fiogbe

**Affiliations:** 1 Centre National Hospitalier et Universitaire Hubert Koutoukou Maga, Cotonou, Bénin; 2 Centre Hospitalier Universitaire de la Mère et de l’Enfant, Lagune, Cotonou, Bénin

**Keywords:** Moelle attachée, incontinence urinaire, imagerie par résonance magnétique médullaire, Tethered cord syndrome, urinary incontinence, medullary MRI

## Abstract

Le syndrome de la moelle attachée est un ensemble de symptômes neurologiques dû à une traction axiale constante ou intermittente du cône terminal de la moelle spinale, fixé en position caudale anormale. Il s'agit d'une lésion congénitale rare dont les symptômes peuvent s'exprimer qu'à l'âge adulte. Nous rapportons un cas clinique chez un garçon de 10 ans découvert à la suite d'une incontinence vésicale et anale qui a été confirmé par une imagerie par résonnance magnétique lombo-sacrée. Il a bénéficié d'une libération neurochirurgicale du cône terminal par un abord postérieur. L'évolution a été marquée par une amélioration des troubles sphinctériens. Ce cas est suivi d'une revue de littérature sur le sujet. Ce cas met l'accent sur l'intérêt de l’imagerie par résonance magnétique (IRM) dans le diagnostic de cette affection.

## Abstract (en)

Tethered cord syndrome is a spectrum of neurological symptoms due to a constant or intermittent axial traction of the terminal cone of the spinal cord, fixed in abnormal caudal position. It is a rare congenital lesion whose symptoms can be observed only in adulthood. We report the case of a 10-year-old boy with tethered cord syndrome discovered due to bladder and anal incontinence and confirmed by lumbosacral magnetic resonance imaging. He underwent neurosurgical release of the terminal cone by posterior approach. Evolution was marked by improvement of sphincteric disorders. This case study has been followed by a literature review on this subject. This case study highlights the role of magnetic resonance imaging (MRI) in the diagnosis of this disorder.

## Introduction

Le syndrome de la moelle attachée est un trouble fonctionnel progressif, provoquée par une fixation pathologique anormale du cône terminal de la moelle spinale à la colonne vertébrale [1]. La croissance différentielle entre la colonne vertébrale et la moelle spinale, il en résulte un étirement, une hypoxie et une ischémie du cône terminal et des dernières racines nerveuses. L'incidence du syndrome de la moelle attachée est évaluée entre 0,05 à 0,25 cas pour 1000 naissances [2] avec une prédominance féminine [3,4]. Il peut être primaire (en rapport avec un filum terminal trop court et trop épais, un lipome sacré intra rachidien, un spina bifida occulta ou une diastématomyélie) ou secondaire (en rapport avec des adhérences conjonctives cicatricielles après la fermeture chirurgicale post natale d'un méningocèle [5]. Le plus souvent, les signes cliniques varient en fonction de l'affection sous-jacente, se révèle dans l'enfance; mais parfois le diagnostic n'est fait qu'à l'âge adulte [6]. L'imagerie par résonnance magnétique est l'examen diagnostic de choix. Elle retrouve le niveau du cône terminal en dessous de L2 et met généralement en évidence la cause de l'attache [7]. Le traitement du syndrome de la moelle attachée est essentiellement chirurgical et consiste en une libération neurochirurgicale du cône terminale par un abord postérieur. Nous rapportons ici un cas de syndrome de la moelle attachée congénitale découverte à la suite d'une symptomatologie urinaire.

## Patient et observation

**Figure 1:**
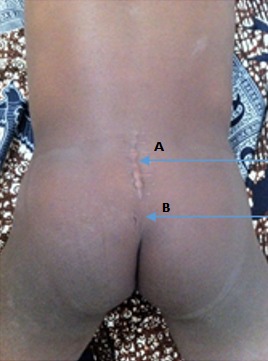
Photo montrant la fossette cutanée de l’enfant: A) cicatrice de l'abord postérieur; B) fossette cutanée en région sacrale

**Figure 2:**
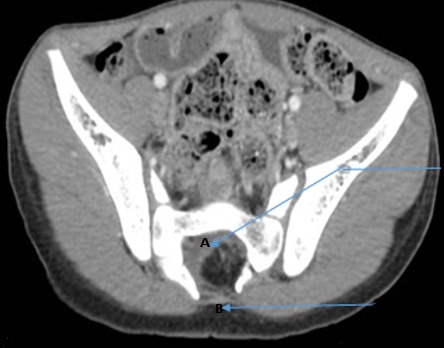
Scanner lombo-sacrée montrant le defect de l’arc postérieur du sacrum: A) tissu graisseux intra rachidien; B) absence de l'arc postérieur de la 3^ème^ pièce sacrée

**Figure 3:**
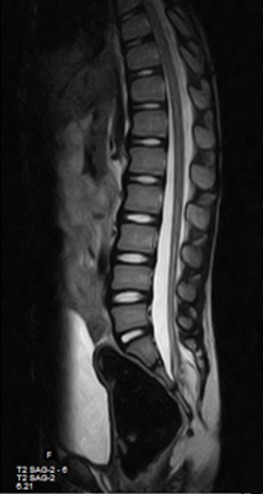
IRM médullaire montrant la lipome intracanalaire accompagné d’un spinabifida (spinalipome) et associé à la moelle fixée en position basse

Il s'agissait d'un enfant de 10 ans, suivi dans la clinique universitaire d'urologie et d'andrologie du Centre National Hospitalier et Universitaire Hubert Koutoukou Maga, Cotonou, Bénin (CNHU-HKM) pour des fuites d'urine. L'interrogatoire avait retrouvé un début à 3 ans de vie par des fuites urines diurnes isolées au cours des efforts de poussées abdominales, évoluant sans rémission pendant 5 ans. Les antécédents médicaux étaient sans particularités. L'examen physique à l'entrée est pauvre; il retrouvait essentiellement une fossette cutanée en région glutéale (Figure 1). Les reflexes étaient normaux et symétriques, il n'avait pas de déficit neurologique. L'échographie de l'appareil urinaire avait permis d'objectiver une vessie diverticulaire sans retentissement sur le haut de l’appareil urinaire. La tomodensitométrie abdomino-pelvienne du 11/09/2014 avait permis de mettre en évidence une absence de l'arc postérieure des 2^ème^, 3^ème^ et 4^ème^ pièces sacrées et une présence de tissu graisseux dans le canal vertébral faisant évoqué un spina bifida avec un aspect de lipoméningocèle sacrée (Figure 2). L'imagerie par résonnance magnétique du 14/11/2014 a permis d'objectiver une formation apparaissant en hypersignal en T1 et T2, en région sacrée à la hauteur de S2, S3 qui était rehaussée par le produit de contraste et apparaissant en intra lombo-sacré en « en doigt de gant» et sur le quel vient s'attacher le filum terminal évoquant une moelle attachée par une formation angiolipomateuse de la région sacrée (Figure 3). L'examen uro-dynamique n'a pu être fait par manque de disponibilité. Le traitement neurochirurgical a consisté à une section du filum terminalis le 05/02/2015. Les suites opératoires étaient normales. Il a bénéficié d'une rééducation périnéale. Il a eu une amélioration du contrôle sphinctérien mais persistance de quelque fuites momentanées à l'effort de poussés.

## Discussion

**Figure 4:**
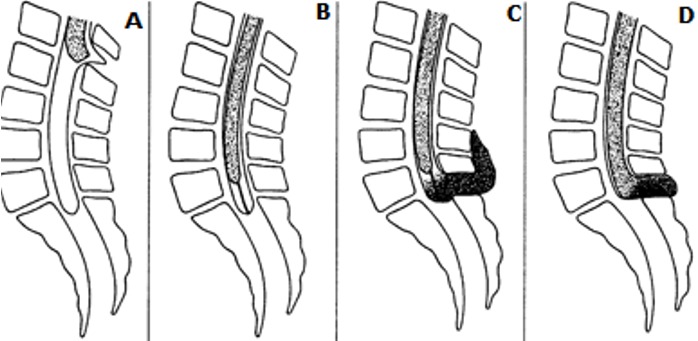
Variétés anatomiques de la moelle attachée in Corcos J: a) spinabifida associé à un sinus dermique mettant en relation les espaces méningées et le plan cutané, moelle fixe en position haute; b) le filum terminal épaissi et attaché empêche l'ascension du cône terminal, moelle fixée position basse; c) lipome intracanalaire accompagné d'un spinabifida (spinalipome) et associé à la moelle fixée en position basse; d) association de spinabifida, méningomyocèle et lipome (lipomyéloméningomyocèle), moelle fixée en position basse

Le syndrome de la moelle attachée est une pathologie rare. Son incidence se situe entre 0,05 et 0,25 pour 1000 naissances [2] et sa prévalence est de 14 pour 1000 enfants énurésiques [3]. Il s'agit en effet d'une affection rare, c'était notre première expérience depuis 3 ans d'exercice de la neurochirurgie à Cotonou. Le syndrome de la moelle attachée peut être associé à des syndromes congénitaux, notamment un syndrome VACTERL dans 39% des cas [8] et un syndrome de Rubinstein-Taybi [9]. Quatre grandes variétés anatomiques de la moelle attachée congénitale ont été décrites [10]: 1) le spinabifida est associé à un sinus dermique, mettant en relation les espaces méningés et le plan cutané moelle fixé en position haute; 2) le filum terminal est épaissi et attaché empêchant l'ascension du cône terminal. Moelle fixée en position basse; 3) le lipome intracanalaire est accompagné d'un spinabifida et associé à la moelle fixée en position basse; 4) association de spinabifida et lipomyéloméningocèle: moelle fixée en position basse (Figure 4).

Elles ont en commun la fixation du cône terminal qui sera donc soumis à des tractions au cours de la croissance ou lors des mouvements répétitifs du rachis. Les symptômes apparaissent au cours de la première enfance, ce qui était le cas de notre patient. Néanmoins chez de nombreux patients, elles n'apparaissent qu'à l'âge adulte [6]. Les anomalies orthopédiques sont retrouvées dans 30% des cas [11]. Sur le plan neurologique le déficit intéresse les membres inférieurs, la musculature périnéale et le sphincter anal dans 50% des cas [12]. Chez notre enfant il s'agissait d'une incontinence urinaire à l'effort et d'énurésie. Le type d'incontinence est rarement précisé, il peut s'agir de symptômes aussi divers qu'une énurésie, une incontinence par impériosité ou une incontinence d'effort [10,12]. Le diagnostic est neuroradiologique. Les clichés du rachis montrent dans 50% des cas un spina bifida osseux d'au moins 2 étages. Avant trois mois de vie, l'échographie montre une fixation anormale de la moelle associée ou non à un lipome. Par definition, le diamètre du filum dépasse 2 mm au niveau de L5-S1 [13]. Au myéloscanner, l'aspect est très évocateur, mais il est actuellement supplanté par l'IRM médullaire qui est la technique de choix pour le diagnostic et le suivi [14]. Nous avions fait un myéloscanner qui permettait de faire le diagnostic sans pourtant pourvoir faire la différence entre les types. L'IRM, nous a permis de faire le diagnostic de moelle attachée associant une spina bifida et lipomyéloméningocèle: moelle fixée en position basse.

L'évaluation du retentissement urologique fait partie du bilan systématique de toute moelle attachée. Des anomalies urodynamiques sont retrouvées dans 93% des cas d'où l'intérêt des explorations dans un but thérapeutique et pronostique [10]. Tous les types d'activités vésicales peuvent être rencontrés: vessie aréflexique, hyper-réflexique, intermédiaire voire normale [15]. La normalité des explorations urodynamiques diminue avec l'âge: 42% des bilans urodynamiques sont normaux avant 18 mois, 21% après 18 mois [12]. L'intervention précoce (avant 18 mois) est marquée par une réversibilité neurologique dans 71% des cas et urinaire dans 83% des cas [16]. Le diagnostic ayant été tardif dans notre cas, l'intervention a été réalisée après l'âge de 10 ans. L'évolution un an après l'intervention, on observe une régression totale de l'énurésie, une régression partielle de l'incontinence d'effort.

## Conclusion

Le syndrome de la moelle attachée est une affection congénitale rare pauci-symptomatique mais dont le diagnostic doit être fait en période néonatale. Il doit être évoqué devant toute anomalie cutanée de la région lombosacrée. L'attitude thérapeutique est discutée. La chirurgie est systématique chez les enfants surtout avant 18 mois. Elle est indiquée uniquement en cas de progression des symptômes chez l'adolescent et chez l'adulte.

## Conflits d’intérêts

Les auteurs ne déclarent aucun conflit d'intérêts.
